# Classically conditioned modulation of pain depends on stimulus intensity

**DOI:** 10.1007/s00221-021-06285-4

**Published:** 2022-02-11

**Authors:** Daniel S. Harvie, Eva Y. Poolman, Victoria J. Madden, Nick A. Olthof, Michel W. Coppieters

**Affiliations:** 1 School of Health Sciences and Social Work, Griffith University, Brisbane and Gold Coast, Australia; 2 Menzies Health Institute Queensland, Griffith University, 170 Kessels Road, Nathan, Brisbane and Gold Coast, QLD 4111 Australia; 3 IIMPACT, Allied Health and Human Performance, University of South Australia, Cape Town, South Africa; 4 Pain Unit, Department of Anaesthesia and Perioperative Medicine, Neuroscience Institute, University of Cape Town, Cape Town, South Africa; 5 Amsterdam Movement Sciences, Faculty of Behavioural and Movement Sciences, Vrije Universiteit Amsterdam, Van der Boechorststraat 9, 1081 BT Amsterdam, The Netherlands

**Keywords:** Classical conditioning, Chronic pain, Psychophysics, Associative learning, Conditioned hyperalgesia, Nocebo

## Abstract

Innocuous cues that become associated with pain can enhance pain. This is termed classically conditioned hyperalgesia. The size of this effect varies under different conditions. We aimed to test whether the sensitising effect of pain-associated cues depends on the intensity of the paired test stimulus. To do this, two virtual reality environments were paired with either painful or non-painful vibrotactile stimuli in a counterbalanced fashion. The differential effect of the two environments was evaluated using pain intensity ratings of paired electrocutaneous test stimuli at three different intensity levels. Forty healthy participants were included in the study; 30 participants experienced sufficient pain during the learning phase and were included in the main analysis. An effect of environment (*p* = 0.014) and interaction between environment and test stimulus intensity was found (*p* = 0.046). Only the most intense test stimulus was modulated by environment. While the effect was small, the results are consistent with the proposition that pain-associated cues may induce hyperalgesia to some degree, under certain conditions. In particular, results highlight the potential relevance of stimulus intensity during and after the initial painful experience. Further attention is needed to comprehensively understand the variables that impact classically conditioned hyperalgesia.

**Supplementary Information:**

The online version contains supplementary material available at 10.1007/s00221-021-06285-4.

## Introduction

Classical conditioning is the process whereby a normally neutral stimulus can develop the capacity to evoke behavioural responses by becoming associated with innately meaningful stimuli (Pavlov 1928). In pain research, this paradigm has been used to understand pain-related fear and its connection to fear of movement (Vlaeyen et al. 1995; Vlaeyen and Linton 2000, 2012). Here, fear is viewed as an innate response to pain, such that, after formation of an association between movement and pain, fear becomes a learned response to movement (Lethem et al. 1983; Hamm et al. 1989; Van Damme et al. 2004; Liu 2011; Meulders et al. 2011; Glotzbach et al. 2012; Moseley and Vlaeyen 2015). The Imprecision Hypothesis proposes a *pain* learning process that may occur in parallel to any learning of pain-related fear (Moseley and Vlaeyen 2015). Here, pain is viewed as an innate response to nociception, such that, after formation of an association between movement and painful nociceptive events, pain becomes a learned response to movement. Moreover, it proposes that other cues, such as environmental contexts or tactile cues associated with nociceptive events, could also contribute to pain.

No laboratory evidence exists that pain can be evoked by an isolated pain-associated cue, although there is evidence that a pain-associated cue can increase pain intensity and lower pain thresholds (Madden et al. 2016). Current data suggest that stimuli associated with nociception can increase pain intensity by 7.4 on a 0 to 100 pain rating scale (Madden et al. 2016). While changes in pain of less than 2/10 or 30% are not considered meaningful in clinical scenarios (Farrar et al. 2001), we hypothesise that effects may be restrained by low ecological validity and other deficits in laboratory methods. Indeed, there is already evidence of variables that may impact the strength of the effect. For example, the magnitude of the effect is predicted by the intensity of painful stimulation during the learning phase (Jensen et al. 2012, 2015; Harvie et al. 2016), likely because greater intensity invokes a stronger ‘learning signal’.

Further potential influencers of classically conditioned pain modulation may be derived from models of perception and well-established learning principles. In the Bayesian framework of perception, perceptions emerge from a best estimate of reality, based on the integration of relevant information from various sources (Knill and Pouget 2004; Meyniel et al. 2015). Here, a signal’s influence on perception (i.e. its relative weight) depends on factors such as its salience relative to other relevant signals. This would predict that a pain-associated cue may have more influence on perceived pain when paired with a painful stimulus of lower intensity, since the pain-associated cue would have more relative weight. Another possible influence is stimulus belongingness, which expresses the principle that certain stimuli may be more likely to become associated because of their functional relevance (Domjan and Galef 1983).

In the current study, we aimed to investigate potential modifiers of the pain-enhancing effect of pain-associated cues. Based on the Bayesian view of perception, we hypothesised that pain-associated Virtual Reality (VR) environments would have their greatest pain-enhancing effect on painful stimuli of lower intensity.

## Methods

### Participants

Participants were recruited through advertisements on a university campus. Participants were not informed of the specific research question or the study hypotheses, but were informed that we were using VR to better understand pain. Participants were eligible if they were healthy, pain-free, and over the age of 18 years. They were excluded if they had a history of chronic (> 3 months) pain, a diagnosed neurological or psychiatric disease, were taking analgesics or psychoactive drugs, were pregnant, or had an electronic implant.

For each participant, the age, sex and questionnaires to assess depression [PHQ-9: Patient Health Questionnaire-9 (Kroenke and Spitzer 2002)], general anxiety [GAD-7: General Anxiety Disoreder-7 (Lowe et al. 2008)] and pain beliefs [FPQ-9: Fear of Pain Questionnaire-9 (McNeil et al. 2018) and PCS: Pain Catastrophizing Scale (Sullivan et al. 1995)] were collected prior to the experiment.

### Experiment design overview

In classical conditioning studies, a *learning phase* pairing a neutral stimulus with an innately response-evoking stimulus is used, in order that the neutral stimulus itself becomes response-evoking—by virtue of its new association with the other stimulus. Following this, a *test phase* is used to measure the degree to which the initially neutral stimulus has acquired the expected response-evoking properties.

**Fig. 1:**
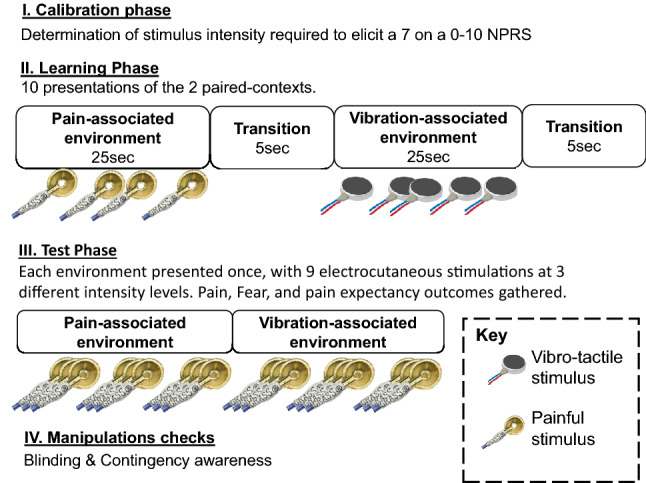
Stages of the experiment

In this study, the *learning phase* (Fig. 1.II) involved pairing two initially neutral VR environments with either painful nociceptive stimuli or non-painful vibrotactile stimuli. The *test phase* (Fig. 1.III) involved assessing the degree to which the pain-associated VR environment modulated pain relative to the vibrotactile-associated VR environment. The specific environment paired with each stimulus was counterbalanced among participants. During the learning phase, each environment was presented 10 times, for 25 s, in a randomised order. In the test phase, the effect of each context on pain sensitivity was tested by presenting electrocutaneous test stimuli in each environment, and asking participants to rate perceived pain intensity.

### Stimulus equipment

#### Electrocutaneous stimulation

Electrocutaneous stimuli were used to deliver painful stimulations. Electrocutaneous stimuli were generated using a Digitimer D185 MultiPulse stimulator (Digitimer Ltd, Welwyn Garden City, Hertfordshire, UK) with 50 µs pulse duration, with a maximum current output of 1.5 A and an inter-pulse interval of 9.9 ms. The stimuli were delivered via Genuine Grass Brand 10 mm gold-plated cup electrodes. The intensity level was varied by altering the number of pulses contained within each individual stimulus. The maximum intensity was a stimulus of approximately 90 ms duration, made up of a train of 9 pulses. The voltage of the stimulations was individually calibrated such that the 9-pulse stimulus would evoke a pain response rated as 7 out of 10 on the Numerical Pain Rating Scale (NPRS); where 0 = no sensation, 3 = first instance of pain, 5 = moderate pain, 7 = significant pain, and 10 = intolerable pain. A staircase method of calibration, with increasing and decreasing intensities (ranging between 10 and 50 V), was performed on the dorsum of the left foot until NPRS 7 was reached three times. These parameters were then applied to the right foot to verify that this was also a NPRS of 7 on the opposite side. Notably, the left foot stimulations were used simply to increase experimental unpredictability, and only pain ratings for the right foot were used in the analysis.

#### Vibrotactile stimulation

Non-nociceptive vibrotactile stimuli were used as the control stimulus. This consisted of a 10 mm, 3 V vibration motor, oscillating at 200 Hz for approximately 500 ms duration. The motors were placed 1 cm medial to the electrocutaneous stimulus on each foot.

#### Environments as conditioning stimuli

VR was used to present different environmental contexts. These were presented using custom VR software (MoOVi—Wearable Computer Lab) on a Windows PC (Alienware 17 R4, P31E, China). Three ‘neutral’ environments were sourced from databases of equirectangular (360°) photographic images. One indoor and one forest scene functioned as the contextual conditioned stimuli (see Supplementary File 1). Based on previous research that showed VR environments do not differentially influence pain, the environments were regarded as neutral with respect to pain at baseline (Smith et al. 2017).

### Procedure

During the experiment, participants were seated in a chair, VR headset in place, and stimulators on both feet. Pain ratings were collected for all stimuli during the third, fifth and eighth environment presentations during the learning phase, so that we could assess if stimuli were sufficiently painful during learning. Calibration was labelled as successful if at least half (5/10) of these stimuli were rated as equal or above NPRS 5 (‘moderate’ pain).

During the test phase, each environment was presented once (Fig. 1.III) in counterbalanced order among participants. Nine electrocutaneous stimuli, with a 10-s inter-stimulus interval, were delivered in each test phase environment. These stimuli were presented at three different intensities (low (1 pulse), medium (3 pulses) and high (5 pulses)) and in block randomised order.

#### Expectancy and fear learning

Expectancy and fear ratings were collected to explore the potential role of expectancy and fear in mediating pain modulation. These ratings were collected at the beginning of each test-phase environment prior to electrocutaneous stimulation. Questions were asked prior to electrocutaneous or vibrotactile stimulation via a digital audio recording. Participants rated the extent to which they expected to receive a painful stimulus on a 0 to 10 US expectancy scale, where 0 = I do not expect pain at all, and 10 = I fully expect to receive painful stimuli. Fear ratings were collected by asking participants how fearful they felt on a scale from 0 to 10 where 0 = not at all fearful, and 10 = extremely fearful.

#### Blinding and contingency awareness

Previous literature suggests that classical conditioning relates strongly to, and may depend on, propositional learning (Lovibond and Shanks 2002; Mitchell et al. 2009). Therefore, participants were asked after the experiment, “*At any time during the experiment, were you able to tell when you were likely to get a painful electrical stimulus?’ If yes, when did you receive them?*” (Fig. 1.IV). Participants were labelled as contingency aware if they correctly identified which environment had been associated with pain during the learning phase*.* Additionally, although participants were naïve to the specific experimental aims at the beginning of the study, we assessed the persistence of blinding after the experiment by asking participants ‘*What do you think we are aiming to test in this study?*’*.*

### Data analysis and statistics

**Fig. 2:**
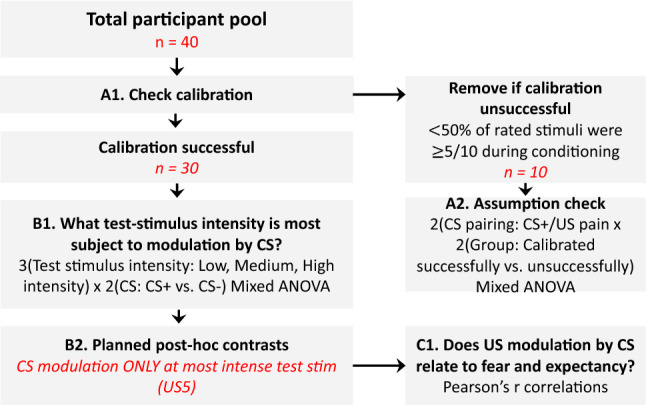
Overview of the study and analysis procedure

For analysis and discussion, the abbreviation CS + (i.e. Conditioned Stimulus) was used to denote the pain-associated VR environment. The abbreviation CS− was used to denote the vibrotactile-associated VR environment. The abbreviation US (i.e. Unconditioned Stimulus) was used to denote the painful stimulus. The analysis plan is represented in Fig. 2, italicised text represents the flow of participants and data.

#### Calibration check (Fig. 2, A1)

Because prior studies have identified pain intensity as an important factor in achieving conditioned hyperalgesia, we planned to exclude cases where calibration did not result in sufficient pain (as defined above) during learning. To confirm that this a priori plan was appropriate, we analysed whether those with unsuccessful calibration (*n* = 10) failed to show classically conditioned hyperalgesia as expected (Fig. 2, A2). As planned, only participants reporting adequate pain intensity during the learning phase (*n* = 30) were used in further analyses. This also fit with the aim of the study to explore factors associated with a larger effect, and their potential to contribute to cumulative effect that is more meaningful.

#### Primary analysis (Fig. 2, B1)

For the primary analysis, we wanted to clarify which test stimulus intensity was most subject to modulation by the CS. For this, we used the test phase data in a 2 (CS: CS + vs. CS −) × 3 (Test stimulus intensity: Low, Medium, High intensity) repeated-measures ANOVA. We planned to then use contrasts to further probe differential classically conditioned effects at the different intensities (Fig. 2, B2). A differential effect of the VR environments was only shown for the high-intensity test stimulus.

#### Secondary analysis

To interrogate the potential relationship between pain and fear, and pain and expectancy, we used two Pearson’s *r* zero-order correlations (Fig. 2, C1). Difference scores calculated by subtracting the scores reported in the pain-associated environment from those in the vibrotactile-associated environment were used for each bivariate correlation.

#### Assumption checks

Prior to analysis, data were checked for normality. Normality was tested using the Shapiro–Wilk test, and the eyeball test and skewness and kurtosis. Additionally, Mauchly’s test of sphericity was used to see if the variances of the differences between all combinations of related groups were equal. Where the sphericity assumption was violated, the Huynh–Feldt correction was used. Tests were performed as two-tailed and the cut-off level for significance was set at *p* < 0.05 for ANOVA testing. Bonferroni corrected *p* values were employed for pairwise comparisons.

#### Reporting

Results were expressed in terms of absolute mean difference between pain during the pain-associated and vibration-associated contexts, as the mean difference expressed as a percentage (relative to the mean of all CS + and CS − ratings). Statistical significance, and the effect sizes Cohen’s *d* and partial eta-squared (${\eta }_{p}^{2}$) were also used where appropriate.

#### Sample size

Our sample size calculation was based on the primary within-subject analysis examining whether the three different test stimulus intensities would be differently modulated by the paired contexts. Although we did not have prior data to inform the calculation, we deemed it important to be able to detect at least a medium effect (e.g. ${\eta }_{p}^{2}$=0.06) with 80% power and alpha set at 0.05. Based on these inputs, we estimated the need for at least 28 participants. Based on previous experiences (Harvie et al. 2016), substantial numbers of participants can report insufficient pain during the experiment despite calibration. Since we planned a priori to exclude such participants, we increased the sample size to 40.

## Results

### Participants

Forty healthy, pain-free volunteers [20 females, mean (SD) age = 29 (7) years] met the inclusion criteria and participated in the study. There were no dropouts. Overall, participants were within normative ranges with respect to psychological scales including the PHQ-9 [3.2 (3.9) = no significant depressive symptoms], GAD-7 [3.5 (3.3) = minimal anxiety], FPQ-9 [21.5 (6.5) = low levels of fear of pain], Pain Catastrophizing Scale [12.3 (7.8) = no clinically relevant pain catastrophizing].

### Calibration assessment

**Fig. 3:**
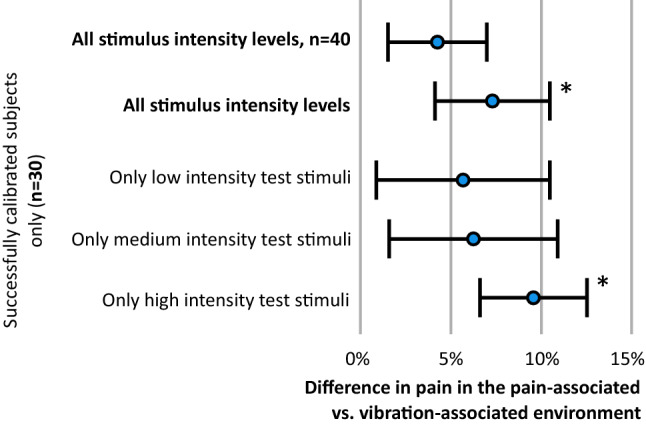
The average percentage difference between CS + /US and CS–US pairings among participants. An asterisk is shown where a significant difference was found between pain- and vibration-associated environments

Of the 40 participants, 10 were classified as having unsuccessful calibration due to experiencing insufficient pain. The ‘Assumption check’ ANOVA found no main effect of CS type (*F*(1,38) = 0.1, *p* = 0.74, ${\eta }_{p}^{2}$=0.00) indicating no significant overall difference in pain ratings between the pain-associated context and the vibration-associated context (Fig. 3). However, there was a significant two-way interaction between calibration success and CS (*F*(1,38) = 5.7, *p* = 0.022, ${\eta }_{p}^{2}$=0.189). Paired *t* tests (with a Bonferroni corrected significance threshold of *p* = 0.025) revealed a significant difference between CSs for the successfully calibrated group only (*t*(29) = 2.6, *p* = 0.015, *d* = 0.22) with a group mean (SD) of 7.3 (17.4)% higher pain ratings in the pain-associated context [mean (SD) NPRS = 3.5 (1.0)] than in the vibration-associated context [3.2 (0.9)] (see Fig. 3). In the unsuccessfully calibrated group, there was no significant effect of CS (*t*(9) = − 1.5, *p* = 0.176). Those who were classified as ‘successfully calibrated’ reported, on average, a pain intensity of 5.92 (0.66)/10 during the learning phase. Those who were not classified as successfully calibrated reported, on average, a pain intensity of 3.73 (0.98)/10. As planned, the main analyses proceeded using the 30 participants with ‘successful’ calibration.

### Primary analysis: which test stimulus intensity is most subject to modulation by the CS?

Among those classified as reaching the ‘successful’ calibration threshold, the low-, medium-, and high-intensity test stimuli were rated as [mean (SD) = 1.8 (0.9)/10, 3.8 (1.12)/10 and 4.6 (1.2)/10, respectively]. The primary analysis revealed a main effect of CS (*F*(1,29) = 6.77, *p* = 0.014, ${\eta }_{p}^{2}$=0.189) and a two-way interaction between CS and test stimulus intensity (*F*(2,58) = 3.3, *p* = 0.046, ${\eta }_{p}^{2}$=101). Planned contrasts (with a Bonferroni corrected significance threshold of *p* = 0.017) revealed a differential classically conditioned effect for the high-intensity test stimuli (*t*(29) = 3.37, *p* = 0.002, *d* = 0.34) corresponding to an 11.4 (25.7)% greater differential effect (mean diff: 0.40 (0.66)/10) in the pain-associated (CS +) context [mean (SD): 4.76 (1.20)/10] than in the vibration-associated (CS −) context [mean (SD): 4.36 (1.14)/10] (see Fig. 3). There was no differential classically conditioned effect for medium intensity stimuli (*p* = 0.174) or low intensity test stimuli (*p* = 0.529).

Following the experimental procedure, 25 participants did not correctly guess the aim of the study and were classified as blinded, and all but seven participants correctly reported the experimental contingencies. The effect of stimulus blinding and contingency awareness on the main finding was examined using a 2 (Blinding: Blind vs. Unblinded) × 2 (CS: CS + vs. CS −) and a 2 (Contingency: Aware vs. Unaware) × (2 (CS: CS + vs. CS −) mixed ANOVA. There was no interaction with either contingency awareness or blinding (all *p* > 0.17).

### Secondary analyses: relationship between the differential classically conditioned effect and fear or expectancy

On average, pain expectancy was greater in the pain-associated context [mean (SD) = 7.5 (3.0)] compared with the vibration-associated environment [1.9 (2.4)]. Similarly, greater fear was reported in the pain-associated environment [mean (SD) fear rating = 4.0 (2.4)] than in the vibrotactile-associated environment [mean (SD) fear rating = 1.6 (1.7)]. The difference in fear between environments appeared to have a weak but significant relationship with difference in pain (*r* = 0.39, *p* = 0.03). This was not true for expectancy ratings (*r* = 0.25, *p* = 0.17).

## Discussion

The hypothesis that pain-associated cues can increase or even provoke pain has gained attention (Madden et al. 2016; Moseley and Vlaeyen 2015). Studies have confirmed that pain-associated cues can enhance pain, but not to a degree that would be considered meaningful in clinical scenarios. Consistent with our previous research, we found that an effect of pain-associated VR environmental contexts was present only under certain conditions (Harvie et al. 2018). That is, an effect of environment was detected only when the reported pain during the learning phase was sufficiently intense. The effect of VR environment was also dependent on the intensity of the painful test stimulus it was paired with—we only found an effect on the higher intensity stimulus.

### Pain as a driver of learning

In classical fear conditioning studies, the more painful the unconditioned stimulus, the fewer trials it takes to establish an aversive emotional association with a previously neutral stimulus (Schafe et al. 2001; Apkarian 2008). Our findings support the suggestion that classically conditioned hyperalgesia also depends on pain intensity during learning, in those only participants meeting our a priori cut-off for sufficient pain intensity showed any effect.

### The role of test stimulus intensity

Based on perceptual models, we hypothesised that pain-associated contexts would have their strongest pain-enhancing effect on painful stimuli of low intensity. Instead, statistically significant modulation in the pain-associated context relative to the vibration-associated context was seen only when the test stimulus was *more* intense (mean difference: 11.4 (25.7)%). The reason why the relationship may be in the opposite to expected direction is difficult to discern from the current data.

### The relationship between fear, expectancy, and pain

It has been suggested that pain-related fear or expectancy may be responsible for classically conditioned hyperalgesia. In our data, only 15% of the variance in pain ratings was explained by fear (*r*^2^ = 0.15), while expectancy explained just 6% (*r*^2^ = 0.06). While the current design precludes inferences of causation, our findings are remarkably consistent with mediation analyses that show that a 14% change in pain can be explained by fear (Meulders et al. 2012).

### Limitations

The major limitation of this study was that, although we calibrated our highest intensity to 7/10, the average reported intensity for that stimulus during conditioning was only 5.4 (1.2)/10. Since high-intensity stimuli may be important to achieving an effect, our study may be limited by the modest intensity of pain reported by participants, even after removal of those reporting the least amount of pain. The need to remove participants not reporting sufficient pain resulted in the further limitation of low participant numbers, which limits confidence in our findings. Among other limitations, participants were not drawn from a patient or pain-susceptible population, although evidence suggests that some individuals may be more susceptible to aberrant pain-related classical conditioning (Harvie et al. 2017).

### Clinical relevance of conditioned hyperalgesia

The belief that pain may be a classically conditioned response is a widespread clinical belief (Madden and Moseley 2016). In clinical scenarios, changes in pain of less than 30% are considered non-meaningful. As such, our study is consistent with the broader literature which has so far failed to prove that clinically meaningful changes in pain can be induced through classical conditioning. Nonetheless, the small effects seen in laboratory studies such as our study may be less than what is possible in clinical scenarios.

## Conclusion

While the effect was small, the results are consistent with the proposition that pain-associated cues may induce hyperalgesia to some degree, under certain conditions. In particular, results highlight the potential relevance of stimulus intensity during and after the initial painful experience. Further attention to understanding the variables that impact classically conditioned hyperalgesia may contribute to determining its potential relevance.

## Supplementary Information

Below is the link to the electronic supplementary material.

Supplementary file1 (DOCX 6437 kb)

## Data Availability

Data have been made publicly available through Harvard’s Dataverse: https://doi.org/10.7910/DVN/MFD7JW.

## Code availability

N/A.
