# Supplementary material for: Classically conditioned modulation of pain depends on stimulus intensity
Source: Exp Brain Res. Author manuscript; Available in PMC 2022 Apr 20. (PMC9015979; doi:10.1007/s00221-021-06285-4)
Supplement: 1784516_Sup_File_1 [file NIHMS1784516-supplement-1784516_Sup_File_1.docx]

**Supplementary File 1**


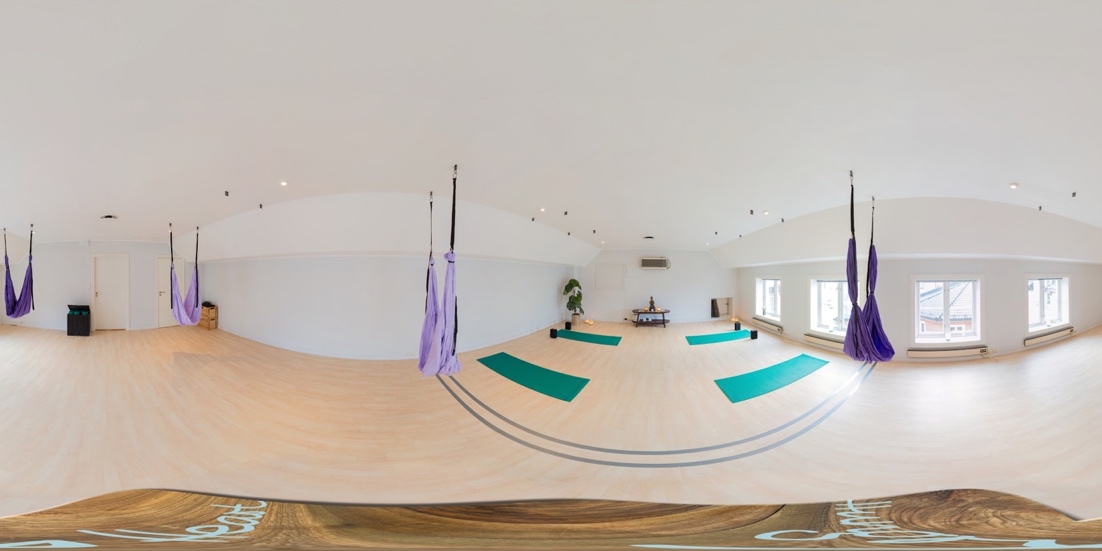


**Figure 1.** The scene considered to have lower belongingness with painful stimuli delivered to bare feet.


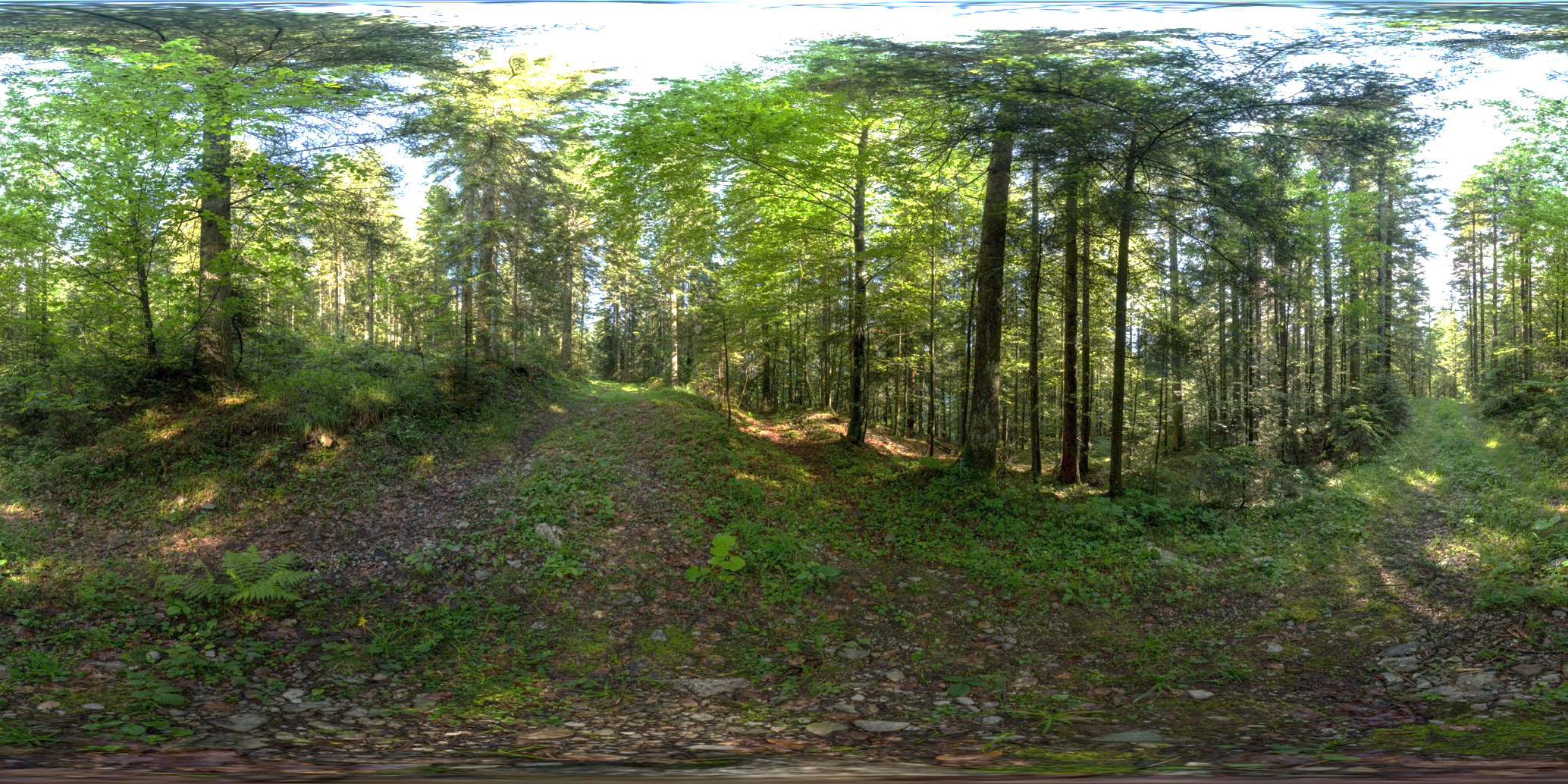


**Figure 2.** The scene considered to have higher belongingness with painful stimuli delivered to bare feet.


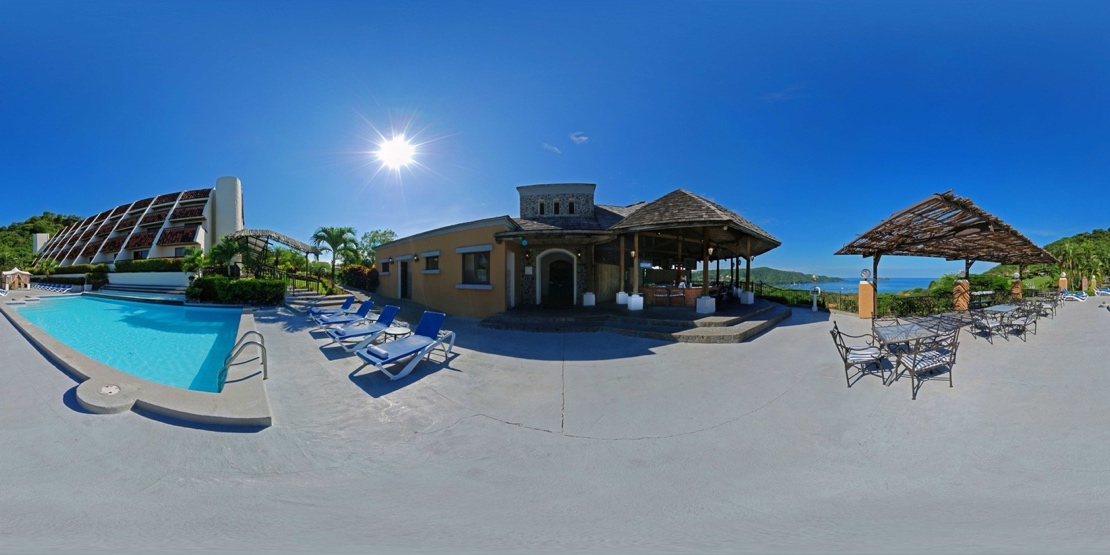


**Figure 3.** The neutral/transition scene, not used in the analysis.
