# Supplementary material for: Classically conditioned modulation of pain depends on stimulus intensity
Source: Exp Brain Res. 2022 Feb 11;240(4):1151–8. doi: 10.1007/s00221-021-06285-4 (PMC9015979; doi:10.1007/s00221-021-06285-4)
Supplement: Supplementary file 1 [file 221_2021_6285_MOESM1_ESM.docx]

**Supplementary File 1**

**Figure 1.** The scene considered to have lower belongingness with painful stimuli delivered to bare feet.

**Figure 2.** The scene considered to have higher belongingness with painful stimuli delivered to bare feet.

**Figure 3.** The neutral/transition scene, not used in the analysis.
